# Resilience of Multi-Layer Communication Networks

**DOI:** 10.3390/s23010086

**Published:** 2022-12-22

**Authors:** Vesa Kuikka, Heikki Rantanen

**Affiliations:** Finnish Defence Research Agency, Tykkikentäntie 1, P.O. Box 10, 11311 Riihimäki, Finland

**Keywords:** network resilience, quality of service, network connectivity, multi-layer communication network, complex network, modelling network structure, system of networks

## Abstract

Advances in the future communication technologies and capabilities of new services in heterogeneous network systems have increased the need for modelling and analysing various aspects of both the resilience of networked systems and usability from the user’s point of view. We extend the traditional network reliability theory to cover a wider scope of quality requirements and applications. The proposed method can be used to model the resilience of different structured networks, and the quality of information services. We use the term resilience to cover both the technical and quality-of-service aspects of user requirements. The modelling method is demonstrated with a use case of a multilayer communication network system. However, the method can be used to model any kind of technological network, such as wireless, sensor, and backbone networks.

## 1. Introduction

High speed, ubiquitous connectivity, massive Internet of Things (IoT), and a huge number of different kinds of sensors are typically identified to be the most important elements of the 5G revolution, where reliable network connectivity would become as critical as electricity, gas, and water for society. As a solution to tight reliability and resilience requirements, the communication network is proposed to be a heterogeneous multilayer network with several connections between different elements of the network. This paper proposes a method to assess the resilience of heterogeneous multilayer networks.

### 1.1. Drivers for Resilient Communication

The war of aggression against Ukraine and its implications for European resilience in terms of EU’s self-sufficiency and economic growth, and maintaining the EU’s standard of living and social and environmental sustainability has been a topic of intensive public debate. Equally, when discussing the resilience of today’s information society, it would collapse without the Internet, and well-functioning and reliable telecommunication networks interconnecting all computers in the world.

### 1.2. Elements of Multilayer Heterogeneous Network

Global data communication networks can be divided into three different networks: core networks, access networks, and increasing numbers of satellite networks are now part of this combined heterogeneous system. A core network, also called a backbone network, is a high-speed, typically optical, network designed to interconnect several access networks. Core networks focus on optimising the performance and reliability of long-distance high-speed data communications.

Access networks, as the name suggests, connect network users to the network, typically using some wireless devices such as mobile phones. Access networks differ depending on the user community. In the military and public safety domain, the reliability of the networks is a key issue. Tactical military networks typically use mobile ad hoc networking (MANET) routing because of the demand for resiliency against network failures. In MANET networks, if some node of the network is lost, a new route is automatically created via other nodes to maintain the connectivity of the network. On the other hand, in commercial mobile networks, a base station failure might block network access in its coverage area [1].

Military communication satellites are typically deployed in geostationary orbit (GEO) positioned at roughly 36,000 km above Earth’s equator, and they rotate in the same direction and speed as those of Earth. GEO satellites provide wide-band and narrow-band communication services, and have been in use for several decades as an elementary part of military communication. GEO satellites offer the ability to stay above a fixed geographic area.

LEO satellites continuously orbit Earth and are not fixed to one point like GEO satellites. Due to this difference, low Earth orbit (LEO) constellations provide very high altitude zones and true pole-to-pole connectivity at all times. LEO constellation system Starlink uses an orbit altitude of 550 km, and satellites achieve a full circle in about 90 min. Due to the low orbit altitude, LEO latency is 30–50 ms (GEO 600–800 ms), being small enough for most real-time applications such as voice.

### 1.3. 3GPP 5G Nonterrestrial Network (NTN) Standardisation Activities

Since 5G standard release 15 in the 3rd Generation Partnership Project (3GPP) standardisation organisation, the feasibility and standard adaptations needed to enable communication via satellite to commercial mobile phones using a normal small antenna have been a topic of intensive work. This is requires much technical effort because Starlink uses rather large user equipment (UE) antennas. The 5G satellite extension is called 5G nonterrestrial networks (NTNs). After the latest 3GPP release, 17, in March 2022, the standardisation work was mature enough for the launch of the first demo satellites at the end of 2022 [2].

U.S. SATCOM project proposal Joint All-Domain Command and Control (JADC2) serves as a good example of how civilian mobile communication technology is adopted by the military domain. JADC2 would use 3GPP 5G NTN standardisation and unmodified user equipment in order to build a private military 5G NTN to serve as a global resilient military satellite network [3].

### 1.4. Network Resilience in 5G Networks and Beyond

Before the introduction of 5G technology an individual user’s service was provided by connecting the user’s device to a dedicated server. Service quality was mainly based on guaranteed SLA (Service Level Agreement) in the IP-based communication network. As a new paradigm, 5G networks are based on SBA (Service Based Architecture), SDN (Software Defined Networking) is used to control data communication, and NFV (Network Function Virtualisation) is mainly responsible for building different kinds of user services and applications. One disruptive concept that will emerge in the 5G era is network slicing. Through network slicing, a single 5G physical network can be sliced into multiple isolated logical networks of varying sizes and structures, dedicated to different types of services [4].

Remembering the above-described development, 5G and other advanced networks are distributed computer networks and the resilience of networks should be evaluated with this in mind. A comprehensive list of resilience disciplines that can be used in data network resilience analysis has been presented in [5].

In this study, we analyse the resilience of multi-layer communication networks using OSI lower-layer link resilience as a parameter. In the context of SDN, our focus is on the data plane rather than on the control plane or the application plane [5]. More enhanced analysis should include also upper layers’ behaviour in order to model the combined resilience of 5G-type networks which is also a topic for future study. The same methodology as in this study can be used to aggregate the effects of different infrastructure layers to calculate the usability of services provided by networked systems [6].

### 1.5. Modelling the Resilience of Networks

The ability of a network to maintain an acceptable level of service in the presence of challenges [7] is a major requirement and design objective. A resilient system continues to offer an acceptable level of service even in the face of challenges. As challenges, and even deliberate attacks, can happen at any information and service infrastructure layer, various detection and protection mechanisms usually co-exist at different levels to mitigate security threats [8].

A process for building resilient computer networks has been presented in [8,9]. The process called $D^{2}R^{2}+DR$ involves the following steps: defend, detect, remediate, recover, diagnose and define. Resilience covers several thematic areas, such as information and network security, fault-tolerance, dependability, performability and network survivability [5,8,9].

One of the research questions asked in [8] is how to compose resilient services driven from a Service Level Agreement (SLA) that describes the desired level of resilience. A great deal of future research is needed to develop a holistic model that would consider various dimensions of describing the resilience of networks. The probabilistic modelling methods of this study provide one possible approach to studying this question in the case of multi-layer communication networks. The same methodology can be applied to modelling systems of connected networks or ‘*connected islands of resilience*’ as described in [10]. Also, based on the same probabilistic definition of resilience, we demonstrate by examples, how node centrality and betweenness measures can be used to analyse and thereafter improve the resilience of network structures and systems of networks.

## 2. Methods of Modelling the Resilience of Heterogeneous Systems of Networks

Our method is based on describing connections within and between networks with a detailed model of links between the nodes in a system of networks. This network model is used as an input to the calculation of resilience values between different parts of the networks. In computer networking, resilience is the ability to provide and maintain an acceptable level of service in the presence of faults and other challenges to normal operation [7,11,12,13]. In this study, we use the concept of resilience in a broad meaning to cover both technical and quality-of-service user requirements. Quality of Service (QoS) is defined as the collective effect of service performance, which determines the degree of satisfaction of a user of the service [14]. It is possible to include only a limited set of requirements in partial models to answer specific research questions. Also, the traditional terms of connectivity and reliability can be used to describe different quality requirements for links in a network structure. Our example is a multi-layer structure of a Backbone network, two Access networks and a satellite system. The methodology is general in the sense that it can describe any number of connected networks.

### 2.1. Extending the Traditional Methods of Modelling Network Reliability

Modelling the resilience of connected communication networks is needed in planning and building more reliable, robust, and usable communication systems and services [15,16]. Functioning connectivity, data transfer capacity and resistance against different disruptive effects in the network structure can be considered in the model. The usability of services from a user point of view can be included in the modelling. Accidental or deliberate actions may lead to deficient network functionality and lower resilience.

We propose a probabilistic model for describing different aspects of resilience between nodes in one communication network, and between different networks of a larger communication system. The model was designed for analysing heterogeneous network systems consisting of several connected networks or heterogeneous structures within one network. Sensor networks, wireless networks, the Internet of Things, and satellite systems are examples of different technologies that could coexist in a system of networks.

Our model is based on the concept of probability of sufficient resilience, an extension of the traditional concept of reliability [17] as the probability of functioning connection. This approach enables defining the quality requirements for selected technical and service-level functions, and then using probabilistic modelling techniques in analysing network structures within one network or connected systems of several networks. Various quality requirements can be defined concurrently on the basis of different research subjects. We present the case study of a multilayer communication system. Our example system of networks consists of a backbone network, two access networks, and satellites.

### 2.2. Modelling Network Structures

We modelled network structures at a detailed level of nodes and links. Directed links between node pairs were assigned individual resilience values 0≤p≤1. To simplify our presentation, we grouped the elements of networks and connections between networks, and assigned them similar properties. In this study, we used only link resilience and bidirectional links, although the theory allows for more general parametrisation: using both node and link properties, and one-directional link resilience values.

**Figure 1:**
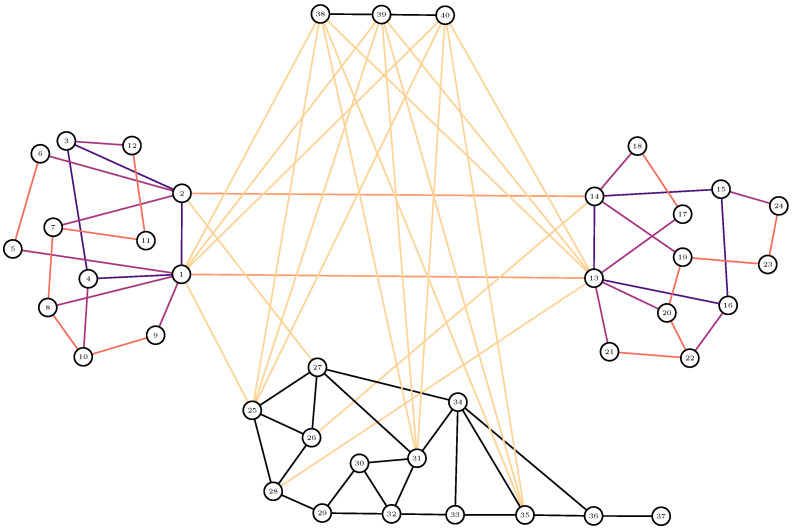
The structure of an exemplary multilayer communication system. The two access networks on the left and right have similar topological structures although their geographical formation may be different. A backbone network is depicted in the lower part of the figure, and a satellite system is in the upper part of the figure.

In our case study, we used the multilayer network structure of Figure 1.

### 2.3. Theory of Network Connectivity and Resilience

In the literature, network connectivity is defined as the average connectivity between all node pairs of a network. Connectivity between two nodes is defined as the probability of a functioning connection between the nodes [18]. In this study, we used a more general concept of resilience, and used the same theoretical probability formulas for resilience as for connectivity.

In the context of resilience, probability can be defined for different purposes depending on the quality requirements in specific situations. Besides connectivity, requirements for data transfer capacity and resistance against cyberattacks or jamming can be used for defining the probability. The probability can be defined by using a complex collection of simultaneous quality requirements. In this study, resilience is defined as the probability of meeting the requirements in a particular scenario. It is also possible to define different concepts of resilience for one scenario by using several concurrent quality requirements. In analysis, these requirements can be categorised and structured as a conceptual network of alternative and mandatory requirements [19]. This may require defining more granular subrequirements, which may be a challenging task.

We assumed that links in a network could be assigned a resilience value. Further, we assumed that link resilience values were independent. If the resilience values between all neighbouring node pairs in the network are known, resilience values between any pairs of nodes in the network can be computed. From the general reliability theory [18], the resilience of a network is

(1) $$R=\sum_{S\in C}\left[\prod_{e\in S}p_{e}\prod_{e\notin S}\left(1-p_{e}\right)\right],$$

where S is a set of links where the network is connected, and C is the set of all connected states of the network. Links are denoted by *e*, and the probability of a functioning link is denoted by $p_{e}$. If all the probabilities *p* are equal, i.e., $p=p_{e}$

(2) R=1−∏h=1E∑s=1h(−1)h−sE−sE−hΦsph,

where $\Phi_{s}$ is the sum of indicator functions, and *s* is the number of nonresilient links. The above equations are polynomials om the order of the number of links *E* in the network. In this form, the equations describe the resilience of an entire network. In our case, we applied the results for pairs of nodes by only taking the relevant terms of Equations (1) and (2). We denote the values of Equation (1) or Equation (2) by $R_{s,t}$ for the resilience between nodes *s* and *t*. We have provided a detailed description and pseudo-code of how to compute the values $R_{s,t},s,t=1...N$ of the resilience matrix in [6]. *N* is the number of nodes in the network. There also is a substantial amount of literature [17,18] on methods to efficiently compute reliability polynomial values that can be applied in computing network resilience values.

A special property of our model is that it accounts for connections via other networks when the resilience values within a network structure (connections between node pairs within a network) are calculated. For example, the resilience of the second access network (right in Figure 1) is improved by connections via the first access network (left in Figure 1) provided that the connections between the access networks are available.

An alternative approach to Equation (1) was presented in [20] where probabilities were computed on the basis of spreading probabilities via self-avoiding paths in a network structure. Self-avoiding paths have no repeated nodes on the path. Examples of self-avoiding paths between two separate networks in Figure 1 are 1-13-14-2 and 2-14-13-1. A pseudoalgorithm for computing the probabilities was provided in Algorithm 2 in [20]. As we simulated the results with two independent methods, testing and validation of the numerical results were on a firm basis. Efficient algorithms that are needed to describe larger network structures were presented in the literature [18].

### 2.4. Definitions of Centrality and Betweenness measures

One way to measure the importance of a node in a given network is to use centrality measures. In this study, we define the centrality of node *s* as the sum

(3) $$R_{s}=\frac{1}{N-1}\sum_{\begin{array}{c} t\in V\\ t\neq s \end{array}}R_{s,t},$$

where *N* is the number of nodes in the network.

We define our measure of betweenness for a node as the relative difference in the cohesion of the network when the node is removed from the network structure, that is,

(4) $$b_{s}=\frac{R-R_{s}}{R},$$

where R is the cohesion of the network defined as

$$R=\sum_{\begin{array}{c} s,t\in V\\ s\neq t \end{array}}R_{s,t}$$

and $R_{s}$ the cohesion of the network with the node *s* removed, that is,

$$R_{s}=\sum_{\begin{array}{c} s,t\in V\backslash \{s\}\\ s\neq t \end{array}}R_{s,t}.$$

## 3. Case Study Results

In this section, we investigate the example network structure of Figure 1. First, in Section 3.1 we study the effects of different levels of resilience between the four networks in Figure 1. Second, in Section 3.2 we study the effects of different levels of resilience within the four networks. The probabilities of sufficient resilience between the networks and within the networks are varied correspondingly. As our main goal was to present modelling methods, we present the case study results in Section 3.1 in more detail than the results in Section 3.2. The model enables varying the link resilience values simultaneously between any neighbouring nodes. This is necessary for many practical planning situations, but our goal here was to illustrate the effects of different factors more distinctly, with simplified scenarios.

We use the following symbols in Table 1 to refer to the four networks and connections between the networks of our case study. Our focus in this study is on the physical-level topology [8,21] or on the data plane of software-defined networking (SDN) architecture [5,22]. The upper layers on the control and application levels [5,8] can be described by the model of this study only partially. In our earlier studies [6,23], we have proposed ideas on how to model the utility of networked services. That will be another dimension that can be added to the multi-layer network model in the future.

**Table 1:** Symbols refer to the four networks in Figure 2. The detailed network structure is depicted in Figure 1. The last two columns show the probabilities of sufficient resilience between neighbouring nodes within the networks (the first four rows) and between the networks (the last six rows) used in Section 3.1 and Section 3.2, respectively.

Symbol	Network	Section 3.1	Section 3.2
a	Access Network a	1.0,0.5,0.25	0.0–1.0
b	Access Network b	0.5,0.25,0.125	0.0–1.0
c	Backbone network	1.0	1.0
d	Satellite network	1.0	1.0
ab	Connections between a and b	0.0–1.0	0.5
ac	Connections between a and c	0.0–1.0	0.5
bc	Connections between b and c	0.0–1.0	0.5
ad	Connections between a and d	0.0–1.0	0.5
bd	Connections between b and d	0.0–1.0	0.5
cd	Connections between c and d	0.0–1.0	0.5

### 3.1. Effects of Connectivity between Networks

**Figure 3:**
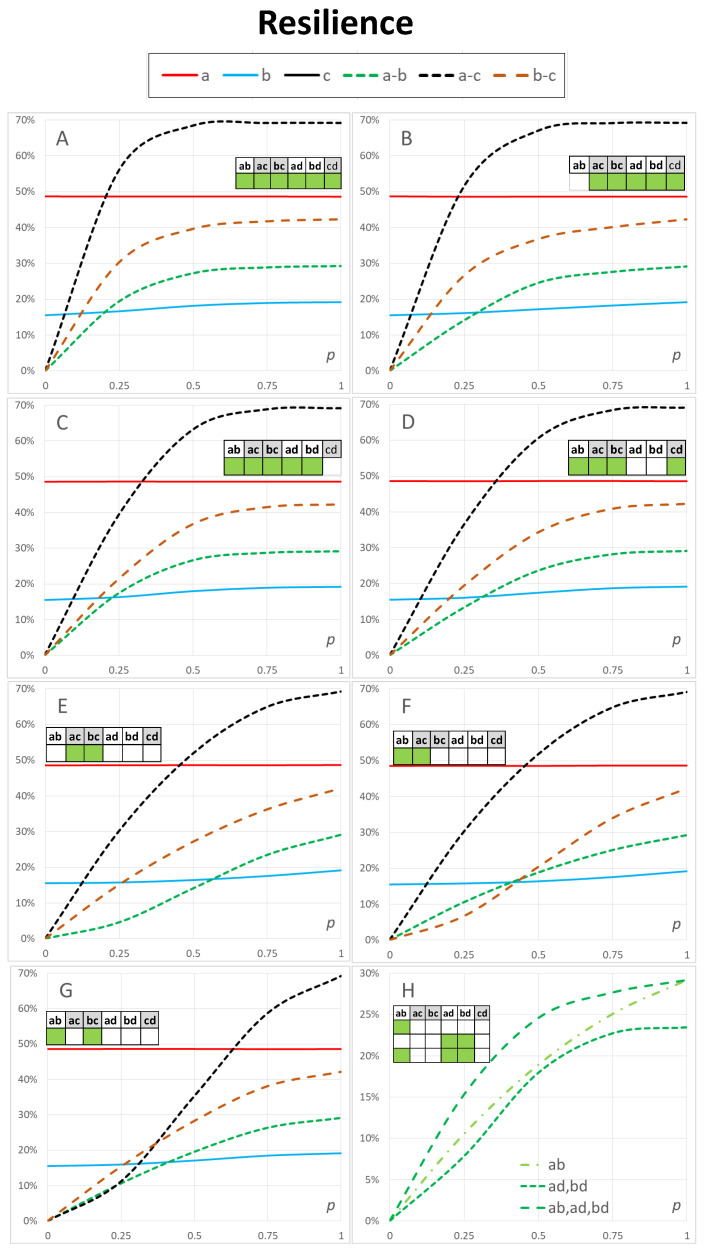
Resilience of the multilayer communication system of Figure 1 with different resilience levels of communication between networks. Resilience values (probability of sufficient resilience) in each network are shown by solid lines a, b and c. Dashed lines a–b, a–c and b–c show corresponding resilience values between different networks. In this figure, *the resilience between nodes within a network is kept constant and the resilience of connections between networks is varied* (horizontal axis).

In this section, we investigate the effects of varying resilience between the four networks of Figure 1. Table 2 shows the connections that are available between the networks in the calculations of Figure 3. For example, the first row of Table 2 indicates that all six types of connections marked as ab, ac, bc, ad, bd, and cd are available. The symbol ab denotes the connections between networks a and b, and so on.

To study the effects of different levels of resilience between the networks, we vary the resilience values of a connection type, keeping the resilience values of connections within the networks constant. For example, in the case of connection type ab, we varied the probability values of the connections between the two node pairs of 1-13 and 2-14 between 0≤p≤1. For simplicity, we kept these values bidirectional, i.e., they were the same for both directions.

Again, to keep our demonstrations manageable, we chose only one set of parameter values for the resilience values of links within the networks. The values are listed in Table 1 for the probability of sufficient resilience of links between nodes in networks a, b, c and d. In this section, we use heterogeneous link resilience values for three types of connections within networks a and b. They demonstrate realistic situations in the case of different technologies, environmental factors, or deliberate interference against network connections. From a theoretical point of view, it is interesting to study two similar topological structures having different communication capabilities or resilience levels.

Table 2 and Figure 3 could be used to investigate different configurations of possible resilience levels between network structures in our case study. Comparing Figure 3A,B shows the drop in the resilience of connections within networks a, b, and c (solid lines, the 100% line for c is not visible in the figure), and the drop in the resilience level between the networks a–b, a–c and b–c (dashed lines). The resilience within networks c and d was not affected by internetwork connections because, within the two networks, resilient connections with p=1 were available (see Table 1 column 3).

The resilience of network *b* shows an increase as a function of *p* in all Figure 3A–G. This difference is more pronounced with low resilience values of resilience values between networks and it disappears when p=1. In addition, Figure 3B shows a small relative drop in the resilience within network *b*. These effects are caused by the two critical access nodes in network *b* with a low resilience value p≈0.5 (see Table 1 column 3 row 2).

In summary, the effects can be different within and between networks. In addition, the effects can be different as a function of the resilience of connections between the networks. In particular, alternative ways of communication are needed in weak communication scenarios. In our example, the two connections via a–b had no effect when other connections provide full resilience when p=1.

Figure 3B–D show different results where connections between the access networks (ab), the backbone network and satellites (cd), and both connections between the access networks and satellites (ad, bd) are not available. Figure 3E shows the results where the connections are only available between the access networks and the backbone network (ac, bc). Figure 3F shows the results where connections were available between the access networks and between the first access network and the backbone network (ab, ac). Figure 3G shows the corresponding scenarios for the second Access network (ab, bc).

One way of comparing Figure 3B–D is to compare the effects of insufficient resilience in connections between access networks (ab), satellite connections between the backbone network (cd), or satellite connections between the two access networks (ad, bd). With high values of connectivity, p≈0.75 the differences were small, as expected. Resilience values for a–c, b–c and a–b are higher in Figure 3C, and a–b is slightly lower in Figure 3B when compared to Figure 3C. The insufficient resilience of connections between the backbone network and satellites cd had major effects on a–c and b–c. Figure 3D shows the corresponding effects of insufficient resilience in both connections between the access networks and satellites. This case had lower resilience when compared to Figure 3B,C, and the effects are similar in a–c, b–c and a–b. When only connections between the access networks through the backbone network (ac, bc) were available (Figure 3E), the relative drop in resilience between the connections between the access networks a–b is most dramatic, especially for low p≈0.25. These changes have intuitive explanations by examining the available and deficient connections.

Figure 3F,G show the very different behaviour of a–c and b–c resilience as a function of the link resilience value *p*. The drop in a–c was larger than that in b–c and a–b is almost unaffected. Access Network a was more resilient than Access Network b (see Table 1, which explains the difference).

We do not present the case (ab, ac, bc), because the results were similar to those in Figure 3D, meaning that the satellites had no improvement in the resilience values when connections were available between the two access networks, and between the two access networks and the backbone network. Satellites also did not affect the case of Figure 3E. The reason for these results is that both the backbone network and the satellite system were resilient with p=1 (see Table 1 Rows 3 and 4 in Column 3).

Figure 3H illustrates how a particular connection type a–b can be compared in different scenarios. Connectivity between the access networks may be an important case, but other cases a–c, b–c, a–d, b–d and c–d are also relevant when the entire communication system is examined. Direct connectivity via satellites may be vital when other connections are not available or their quality is poor.

Figure 3H shows results for connections a–b where only the following connections are available: between the access networks (ab), between each access network and satellites (but not between access networks) (ad, bd) and between access networks, and both access networks and satellites (ab, ad, bd). The results reveal details that were caused by the complex structure of the networks, not evident without modelling and calculations. Satellite connections provide almost the same resilience for connectivity between the access networks when p≈0.5 but lower resilience when p≈0.25 and p≥0.75. This is an example of results that are specific to the network structure in Figure 1 and the parametrisation in Table 1.

We only provide the results of Figure 3H for the resilience of connections between the access networks a–b because, in these cases, all network resilience values a, b and c were constant as a function of *p*.

### 3.2. Effects of Connectivity within Networks

In the previous section, we studied the effects of the resilience of connections between different networks in a networked system on inter- and intranetwork resilience values within a network and between networks, respectively. We varied the internetwork link resilience values and studied the average effects on different network structures.

Here, we investigate the resilience within a network and between networks by varying the intranetwork link resilience values and study the average effects on the internal network resilience and the resilience of connections between two networks in the system. Similarly to the previous section, we used 50% reduced link resilience values for Access Network *b*. Consequently, the link resilience values varied between 0≤p≤1 and 0≤p≤0.5 within Networks a and b respectively.

We varied the resilience values within the four networks of Figure 1 and kept the internetwork link resilience values constant. The link resilience values within the backbone network and satellite system are still kept as fully resilient with p=1.

**Figure 4:**
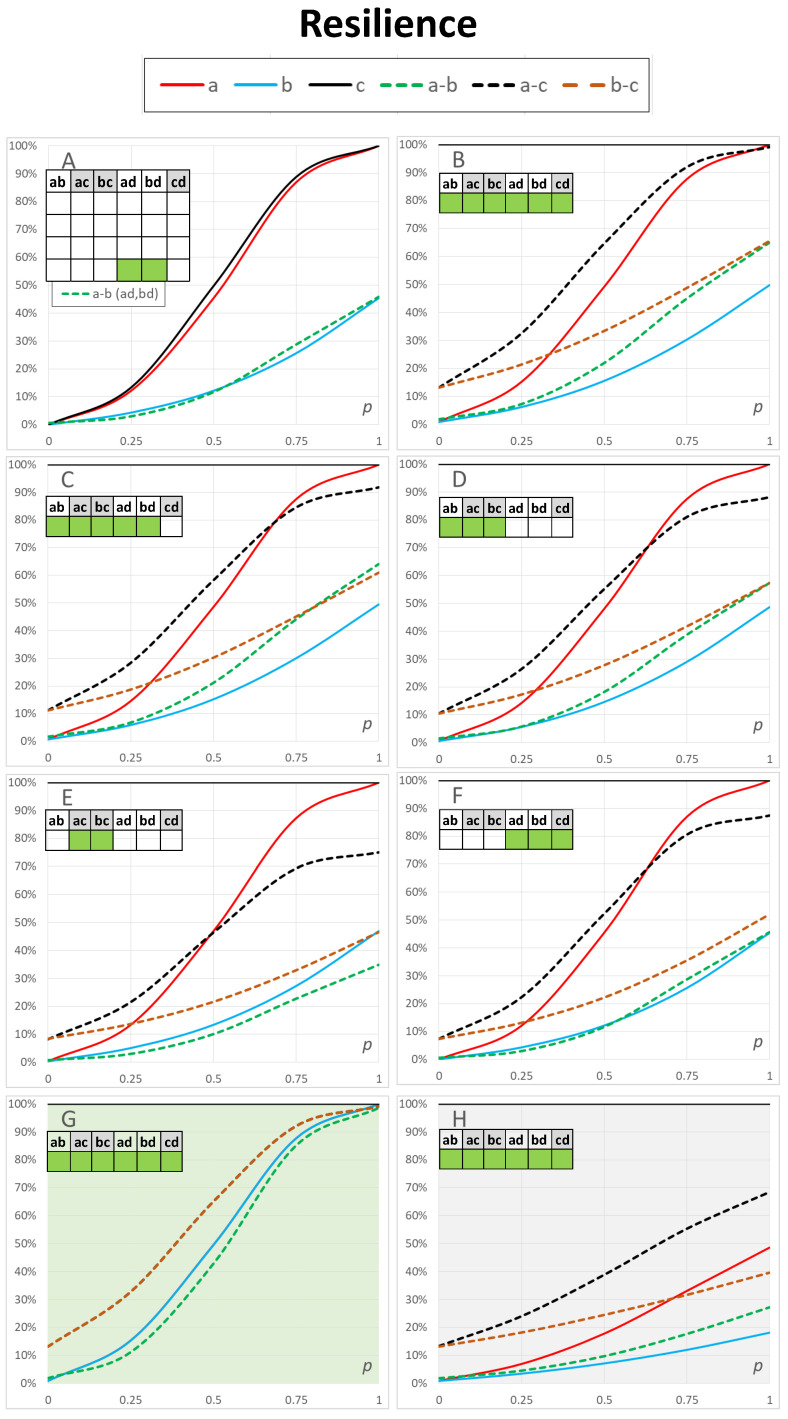
Resilience of the multilayer communication of Figure 1 with different resilience levels of communication within networks. Resilience values (probability of sufficient resilience) in each network are shown by solid lines a, b and c. Dashed lines a–b, a–c and b–c show corresponding resilience values between different networks. In this figure, *the resilience between networks is kept constant and the resilience of connections between nodes in each network is varied* (horizontal axis). (**G**,**H**) are explained in the text.

A relatively low value p=0.5 for link resilience values between networks was used to demonstrate the method (see Table 1 column 4). In practice, these values were estimated in real-world scenarios and according to the actual resilience properties within the networks. For example, environmental circumstances and connectivity can have local changes in wireless networks or sensor networks. Table 3 shows the available connections between the networks in the calculations of Figure 4.

Solid lines in Figure 4A show the basic results of the resilience of separate Access Networks a and b and Backbone Network c. In these calculations, the three networks were not connected with other networks. The resilience values were higher when connections to other networks were available. This can be seen, for example, by comparing Figure 4A,B.

The results for internal network resilience values for networks a, b, and c are slightly different in Figure 4A–F depending on the available connectivity to other networks. One notable effect of these figures is that the resilience values between the access network and the backbone network a–c and b–c had positive values when p=0. This was due to the fact that there was also a single-point connection in this case because we assumed that nodes were still functional. The model enables describing variable resilience values for both element types, links and nodes, but in this study, we investigated only the effects of link resilience. The results in Figure 4B–F can be analysed with the same methods as we did in Section 3.1 where we examined Figure 3. We do not present this analysis here in more detail.

For comparison, Figure 4G shows the case where Access Networks a and b had similar internal link resilience values in the range 0≤p≤1. In the figure, the blue solid curve depicts both a and b, as they had similar properties of network structure and link resilience values. In this case, curves a–c and b–c were also similar, and they are depicted as a brown dashed curve in Figure 4G. The curve for a–b is lower because the access networks were not fully resilient, whereas Backbone Network c was resilient with link resilience p=1.

Figure 4H depicts the case where Access Network a had three types of links with resilience values p,p/2 and p/4, and Access Network b with resilience values p/2,p/4 and p/8. This parametrisation had a dramatic effect on both a and b network resilience values and internetwork a–b, a–c and b–c resilience values in Figure 4H. This example demonstrates a scenario where resilience is very poor except among the four core nodes 1,2,3 and 4 in Figure 1 in Access Network a.

### 3.3. Betweenness and Centrality Results

Next, we demonstrate how centrality and betweenness measures [24] can be used to investigate node-level properties. We present two examples: one from Figure 3A and one from Figure 4B. In order to analyse other scenarios in Figure 3 and Figure 4 calculations with corresponding resilience values between networks and within networks should be accomplished. Our definition in Equation (3) for the centrality measure and Equation (4) for the betweenness measure consider the entire network structure of Figure 1. Centrality values are used to discover the important nodes in the network, and betweenness values are used to discover nodes that are important mediators between other nodes in the network. These nodes are potential critical points or bottlenecks in the network structure, and they should get special attention in strengthening the resilience of the network. As these metrics describe different characteristics of nodes in the network structure, the two metrics values behave differently, albeit they usually are correlated. Also, the vertical scales are different because of their different mathematical definitions.

**Figure 5:**
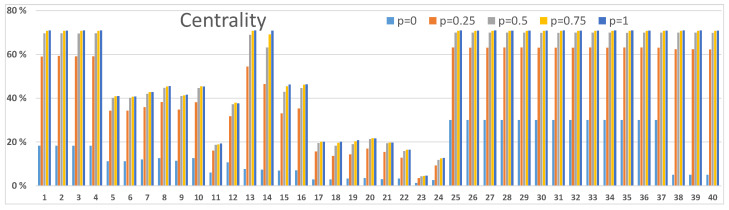
Centrality values of nodes in the network structure of Figure 1. Connections ab, ac, bc, ad, bd and cd between networks are functioning with resilience values p=0,0.25,0.5,0.75 and 1. The results correspond to Figure 3A.

**Figure 6:**
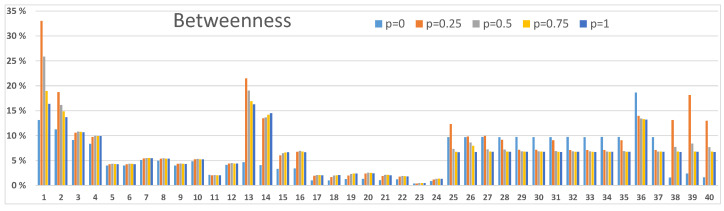
Betweenness values of nodes in the network structure of Figure 1. Connections ab, ac, bc, ad, bd and cd between networks are functioning with resilience values p=0,0.25,0.5,0.75 and 1. The results correspond to Figure 3A.

**Figure 7:**
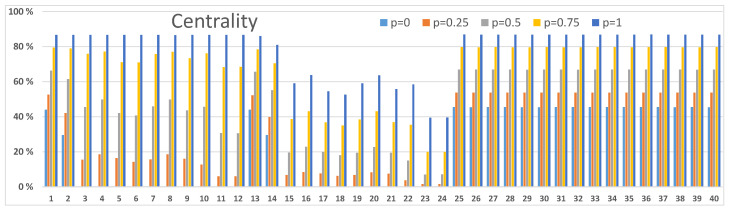
Centrality values of nodes in the network structure of Figure 1. Connections within networks are functioning with resilience values p=0,0.25,0.5,0.75 and 1. The results correspond to Figure 4B.

**Figure 8:**
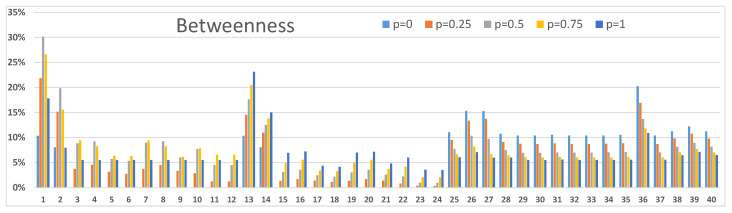
Betwenness values of nodes in the network structure of Figure 1. Connections within networks function with resilience values p=0,0.25,0.5,0.75 and 1. The results correspond to Figure 4B.

The network structure of Figure 1 can be seen in Figure 5, Figure 6, Figure 7 and Figure 8. Nodes 1–12, 13–24, 25–37 and 38–40 compose the two access networks, the backbone network and the satellite network respectively. Both centrality and betweenness values vary in a complex manner, as a result of the network structure, as a function of resilience values *p* between networks in Figure 5 and Figure 6 and within networks in Figure 7 and Figure 8. For example, nodes 1–4 in Figure 5 have similar centrality values because links between them have high resilience values p=1. Lower resilience values p=0.5 between nodes 13–16 have a consequence that nodes 15 and 16 have lower centrality and betweenness values than node 13 and 14 respectively.

Another effect is that the values vary as a function *p*. Betweenness values, as they are defined in this study, can have maxima as a function of *p* as can be seen in Figure 6 and Figure 8. The betweenness measure is particularly useful in detecting nodes and corresponding links incident on those nodes where resilience could be improved by different actions, for example, constructing alternative connections.

We notice from Figure 6 that nodes 1 and 13 in the access networks, particularly when p≈0.25, have a high betweenness value. In the backbone network node 36, particularly for low *p* values, has a high betweenness value. Nodes 38,39 and 40 have high betweenness values for p≈0.25. The mentioned nodes are also central in the network structure as can be seen in Figure 5.

Our second example in Figure 8 shows somewhat different betweenness results. Nodes 1 and 13 have high betweenness values with the higher values of p≈0.5 and p=1 respectively. Node 36, again, has a high betweenness value for low *p* values. Betweenness values of satellite nodes 38,39 and 40 have maxima for lower values of *p* than in Figure 6 and their role is not particularly important anymore.

## 4. Discussion and Contributions

The methods of this study can help in planninf communication systems where the robustness and resilience of different structures in the system of one network or several networks need to be considered in different scenarios. The novel idea of this work is that, in a scenario, any set of elements and connectivity or resilience between these sets can be studied whether they constitute a separate network or not.

The method is based on modelling individual communication links, both intra- and internetwork links in the network structure, and assigning resilience values to each link. The method enables computing resilience for various network structures and connections between these defined structures. Resilience is defined as the probability of a sufficiently resilient link or the probability of sufficiently resilient connections within a network or between networks. The application area of the model can be extended by defining the probability from different viewpoints. For example, we can define the probability of attaining sufficient capacity for data transfer, the functioning of connection as a percentage in a time window or even having the capability of performing a successful operation or task in a scenario.

In summary, the contributions of this study are:

1 We extended the traditional theory of modelling the reliability and functioning of network connections [3] by defining the concept of sufficient resilience. This can be defined more freely in specific scenarios to cover different technical and functional quality requirements.
2 We proposed a detail-level network model describing link resilience. Resilience within or between any collection of nodes in the network structure can be computed on the basis of a definition of a resilient communication link. Links were assumed to be statistically independent, which enabled the use of mathematical formulas of traditional network theory.
3 The model considers connections within a network structure, for example, a subnetwork or a connected network in a system of networks, and connections between network structures when resilience values within or between the network structures are computed. This method provides useful results because, in practice, the concept of resilience should take into account the entire system, affecting the probability of conducting successful computer-aided operations.
4 We proposed an alternative model [25], the network spreading model, based on modelling spreading processes via self-avoiding paths in the network structure. This model and the classical network connectivity model provide similar results. Extensions of the spreading model can be used to model various spreading processes in the network and thus enable more applications of the methodology. In addition, new ideas in the context of the spreading model can be used for studying the resilience of networked systems. On the other hand, classical network reliability theory can have new interpretations in many other application areas of spreading models in social, biological and technological network analyses [19,20,25].
5 We have shown by example how analysing centrality and betweenness measure results together with network resilience calculations can be used to locate important nodes and links in the network structure whose enhanced resilience, for example by building a parallel connection, would probably improve and maximise the overall network resilience.

## 5. Conclusions

We presented a methodology for describing the resilience of a system consisting of several connected networks. Requirements for the resilience and usability of services can be defined or delimited case by case depending on the research question.

The classical network reliability model and the spreading model with self-avoiding paths are two alternative approaches to calculating resilience values for a network structure or connections between two networks in a system consisting of several networks. The network topology of a network structure is modelled on a detailed link and node level.

A multilayer communication network system is used as a case study. The example network system consists of a backbone network, two access networks and a satellite system. The model considers the system of several networks as one network structure permitting the computation of resilience between all the elements or structures in the system.

Practical applications of the model are analysing the resilience of individual networks (separately or as a part of the networked system of several networks), analysing the resilience of connections across networks, analysing different technologies, comparing topological network structures, etc. In addition, the model can be used to investigate the resilience of any individual component, link, node or set of components, of the network. This information can be used to plan and build more resilient and usable system configurations.

The methods of this study can help in planning and building more resilient communication networks and high-quality services. Different aspects of user requirements can be considered in analysing larger systems of several connected heterogeneous networks.

## Figures and Tables

**Figure 2:**
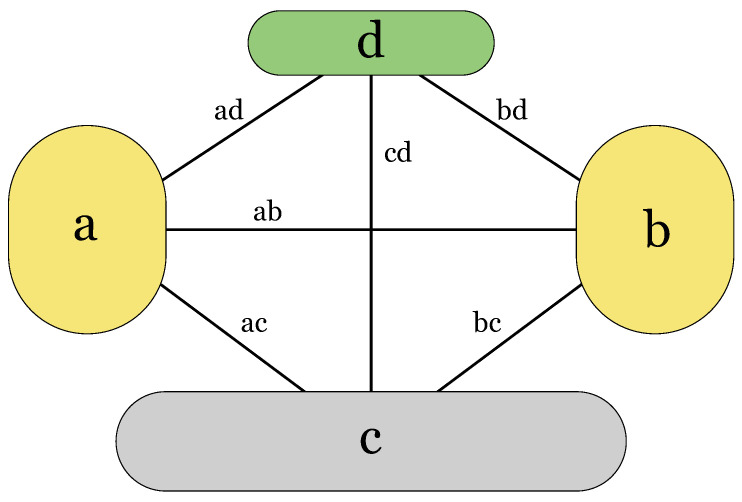
System of connected networks in our case study. Notations correspond to Table 1, Column 1.

**Table 2:** Availability of connections between networks a, b, c and d in Figure 3.

Figure	ab	ac	bc	ad	bd	cd
Figure 3A	y	y	y	y	y	y
Figure 3B		y	y	y	y	y
Figure 3C	y	y	y	y	y	
Figure 3D	y	y	y			y
Figure 3E		y	y			
Figure 3F	y	y				
Figure 3G	y		y			
Figure 3H	y					
Figure 3H				y	y	
Figure 3H	y			y	y	

**Table 3:** Availability of connections among networks a, b, c and d in Figure 4.

Figure	ab	ac	bc	ad	bd	cd
Figure 4A						
Figure 4B	y	y	y	y	y	y
Figure 4C	y	y	y	y	y	
Figure 4D	y	y	y			
Figure 4E		y	y			
Figure 4F				y	y	y
Figure 4G	y	y	y	y	y	y
Figure 4H	y	y	y	y	y	y

## Abbreviations

The following abbreviations are used in this article:

3GPP	3rd Generation Partnership Project
5G	Fifth Generation
5G NTN	5G Non terrestrial networks
EU	European Union
GEO	Geostationary Orbit
IoT	Internet of Things
IP	Internet Protocol
JADC2	Joint All-Domain Command and Control
LEO	Low Earth Orbit
MANET	Mobile Adhoc Networking
NFV	Network Function Virtualisation
NTN	Non-Terrestrial Network
OSI	Open Systems Interconnection
QoS	Quality of Service
SATCOM	Satellite Communication
SBA	Service-based Architecture
SDN	Software-Defined Networking
SLA	Service-Level Agreement
UE	User Equipment
